# Brain Atrophy Estimated from Structural Magnetic Resonance Imaging as a Marker of Large-Scale Network-Based Neurodegeneration in Aging and Stroke

**DOI:** 10.3390/geriatrics2040034

**Published:** 2017-11-10

**Authors:** Michele Veldsman

**Affiliations:** 1 Nuffield Department of Clinical Neurosciences, University of Oxford, Oxford OX3 9DU, UK; michele.veldsman@ndcn.ox.ac.uk; Tel.: +44-790-809-0888; 2 The Florey Institute for Neuroscience and Mental Health, University of Melbourne, Melbourne VIC 3084, Australia

**Keywords:** stroke, aging, atrophy, brain structure, network connectivity, neurodegeneration

## Abstract

Brain atrophy is a normal part of healthy aging, and stroke appears to have neurodegenerative effects, accelerating this atrophy to pathological levels. The distributed pattern of atrophy in healthy aging suggests that large-scale brain networks may be involved. At the same time, the network wide effects of stroke are beginning to be appreciated. There is now widespread use of network methods to understand the brain in terms of coordinated brain activity or white matter connectivity. Examining brain morphology on a network level presents a powerful method of understanding brain structure and has been successfully applied to charting the course of brain development. This review will introduce recent advances in structural magnetic resonance imaging (MRI) acquisition and analyses that have allowed for reliable and reproducible estimates of atrophy in large-scale brain networks in aging and after stroke. These methods are currently underutilized despite their ease of acquisition and potential to clarify the progression of brain atrophy as a normal part of healthy aging and in the context of stroke. Understanding brain atrophy at the network level may be key to clarifying healthy aging processes and the pathway to neurodegeneration after stroke.

## 1. Introduction 

Network-based methods are increasing in popularity as a means to understand the complex functioning and wiring of the human brain. The dominant methods—functional and structural connectivity—are designed to estimate coordinated brain activity and the complex white matter wiring of the brain, respectively. Connectivity and network methods can also be based on shared morphometric features such as grey matter volume or cortical thickness. While not as popular relative to methods that estimate functional or white matter connectivity, these methods have the potential to reveal fundamental principles of brain development and degeneration [1]. Networks derived from different MRI modalities, such as diffusion MRI, morphometry and functional MRI, show similar properties, spatial topographies [2,3] and patterns of disruption in neurological disease [2,4].

As network analysis methods have developed, analysis of atrophy in aging and neurological disease has advanced from purely descriptive topography, to more formal estimates of changes to network properties of distributed anatomical regions at finer scales [2]. Morphometry-based network and connectivity methods can be broadly divided into voxel- or vertex-wise approaches and region of interest approaches that utilize either volumetric- or surface-based estimates of cortical morphology (see Evans, 2013 for a fuller taxonomy of structural methods).

A large body of evidence has identified distributed regions across the brain that show atrophy in the course of healthy aging [5,6,7]. This may reflect natural degeneration within large-scale functional networks [8]. This atrophy may be accelerated or aggravated by stroke where, despite typically focal damage, there are network wide effects on the brain [9,10,11]. Evidence from dementia subtypes show that neurodegeneration appears to target intrinsic functional networks, with patterns of atrophy closely resembling healthy functional networks and networks based on structural covariance [4,12]. A similar picture is emerging of network-based neurodegeneration in the course of healthy aging that is accelerated after stroke. Tracking structural covariance network changes in aging may inform theories of intrinsic functional network degeneration in the same way that these methods have informed theories of intrinsic functional network development [13] and illuminate the pathway from stroke to accelerated neurodegeneration. This review will focus on structural network methods that allow for reliable estimates of brain morphology, and review the emerging literature showing network neurodegeneration in healthy aging and after stroke.

## 2. Measuring Brain Morphometry from Magnetic Resonance Images

Clinicians routinely consult structural brain images in the assessment of aging and neurodegenerative disease with the understanding that brain atrophy is a macroscale indicator of neurodegeneration. Although atrophy can often easily be detected by eye in the course of neurodegenerative dementias, more subtle changes to brain morphometry, often in presymptomatic stages, require quantitative methods for detection. Brain regions can be segmented according to known anatomy, either manually, automatically or with varying degrees of manual intervention. Features of a brain region’s morphometry, such as shape or volume, can then be estimated either across or within individuals. Although manual segmentation remains the gold standard, it is impractical for large datasets, segmentation of multiple regions [14] or estimation of cortical thickness from the complex folding of the cortex. Automated methods allow multiple regions to be delineated and analysed simultaneously, which is ideal for large and longitudinal datasets. Automated methods also allow parcellation of the brain into hundreds of regions, allowing connectivity between these regions to be estimated. Reassuringly, there is high correspondence between automated methods and manual segmentations [14,15]. Automated methods can be broadly divided into volume-based and surface-based methods. Volumetric measures estimate tissue volume, which combines tissue surface area and thickness. Surface area and thickness may change independently and at different rates, which cannot be easily captured by composite volumetric measures. In contrast, surface measures allow these facets of tissue structure to be examined independently. Regardless of the method used to estimate the morphometric feature, changes in that feature over time, or differences across populations, network methods can be applied to formally quantify the relationship between regions across the brain. Before network or connectivity methods can be applied, morphometric measures must be estimated on a voxel-, vertex-, or region of interest-wise level. These methods are outlined below.

### 2.1. Voxel-Based and Surface-Based Morphometry 

A two-dimensional image is broken up into smaller units called pixels. In a similar way, a three-dimensional structural image obtained from magnetic resonance imaging (MRI) can be broken up into smaller elements called voxels (volumetric elements). Voxel-based morphometry estimates volume differences or changes on a voxel-wise basis across the brain. Individual subject images are normalized into a common template space so that volume changes can be averaged across individuals and compared across groups. Images are first segmented into grey matter, white matter and cerebrospinal fluid, according to their relative image intensities. The tissue of interest, typically grey matter, is then selected for further processing, including smoothing and linear or non-linear registration to allow for comparison across subjects [1,16]. Parametric statistical tests can be conducted at every voxel [16], producing statistical maps showing the spatial pattern of local and global grey matter changes across time or groups. Voxel-wise statistical models can be corrected for confounding variables such as age, sex and head size.

Structural images are non-linearly transformed to a template space in deformation-based morphometry and the deformation fields used for the transformation are analysed [1,17]. This approach is likely more sensitive to local morphometric changes than VBM, but can also be combined with VBM as an optimized form [1]. Images with high signal-to-noise and contrast-to-noise ratios are important for the accurate estimation of volume density, as is the absence of image artefacts, such as those that result from head motion [1,18].

Volume-based methods do not capture the complex shape and cortical folding of the cortical surface. In contrast to volume-based methods, surface-based methods model the boundary between the inner white matter or the outer pial surface and the grey matter surface, accounting for the complex folding of the cortical surface [19]. Highly accurate models of grey and white matter surfaces are constructed, and the distance between these surfaces is calculated in order to give a measure of cortical thickness [19,20] Deformable models are used to non-linearly align individual surface anatomy to a template space, although surface measurements can also be estimated in native space [21]. Because they account for the complex folding patterns of the cortex, surface methods have better inter-individual alignment, which aids comparison across individuals. Cortical thickness measurements can be made with submillimetre accuracy, allowing enough sensitivity to track changes, such as gradual cortical atrophy, within or across individuals [20]. Surface-based methods allow for estimates of cortical thickness, shape, surface area and gyrification. Surface-based methods have been evaluated against histology and manual segmentation, further validating their accuracy [22].

Surface- and volume-based methods can be applied cross-sectionally to estimate brain atrophy, but the development of longitudinal methods have allowed for more accurate estimation of changes in brain morphometry that accounts for across-subject variability in cortical thickness measures. Longitudinal measures typically use each individual as their own control, often creating a template based on the average of all timepoints from which to register images obtained at each timepoint [23]. Longitudinal designs are more sensitive to morphology changes than cross-sectional designs, and they require fewer participants in order to detect an effect [22,24].

In region of interest-based approaches, the brain can be parcellated into anatomically or functionally specific areas and the morphometric measure can be extracted [25]. The relationship between these regions can then be estimated, using network methods such as graph theory. Graph theory is a branch of network science that formally describes the relationship between different nodes, for example anatomical brain regions, in a network. Region of interest approaches require apriori parcellation of the cortex, which may introduce bias depending on the parcellation scheme implemented. Different parcellation schemes have been shown to change structural network properties [21].

### 2.2. Connectivity and Network Based Methods 

The application of connectivity and network methods to brain morphometry is based on the observation that regions of the brain co-vary in morphological features [25,26]. Regions of the brain that show inter-individual differences in some measure of morphology, co-vary with other regions showing inter-individual differences [26]. Regions with shared morphological features seem to also be part of networks that share functions, such that structural covariance networks closely resemble intrinsic functional connectivity networks [26]. This feature of brain organization has been shown at post-mortem within visual and motor networks [26], emphasizing the link between shared morphometry and shared function. As is the case with functional connectivity, the relationship in morphometry between spatially distributed regions in the brain is not reliant on direct white matter connections [21]. There is however evidence for covariance amongst regions with direct white matter connections, for example in the language network [26].

**Figure 1:**
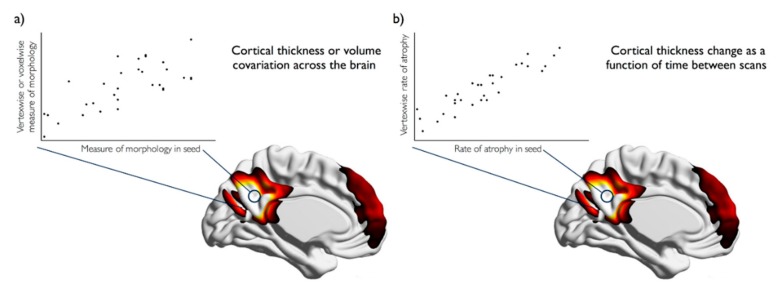
Hypothetical, illustrative examples of (**a**) structural covariance and (**b**) atrophic covariance of the default mode network. (**a**) In structural covariance, volume or cortical thickness in a posterior cingulate seed (circle shape) is compared to volume or cortical thickness with the rest of the brain on a voxel- or vertex-wise basis and at a group level; (**b**) In atrophic covariance, the rate of atrophy in the posterior cingulate region (circle shape) is compared with the rate of atrophy in the rest of the brain on a voxel- or vertex-wise basis and at a group level. Colour spectrum represents strength of correlation, with lighter colours reflecting higher correlation values.

The dominant method for examining relationships in morphometry across the brain is structural covariance. Structural covariance networks can be derived using the same methods applied to derive networks of functional connectivity. The main approaches are seed-based, data-driven and graph analysis [2,26]. In seed-based approaches, the morphological measure, such as cortical thickness or volume, is extracted from the seed region and compared to the morphological measure across the rest of the brain (Figure 1a). Data-driven methods, such as principal or independent components analysis, do not require the selection of an apriori seed region of interest, but instead reduce inter-regional covariance amongst brain regions to components that explain the majority of the variance across people. Finally, graph analysis assigns network properties to structural covariance networks by examining the relationship and correlations in a morphometric measure, between nodes, regions of interest, voxels or vertices. Structural covariance networks can be compared cross-sectionally or longitudinally in order to estimate atrophy within networks in aging or after stroke.

## 3. Network-Based Neurodegeneration in Aging 

Grey and white matter atrophy is a normal part of healthy aging [27]. The pathological basis of this neurodegeneration, as indicated by post-mortem studies, is likely to be the loss of neuropil (i.e., dendritic and synaptic density), rather than neuronal loss [28,29]. Cortical thinning is widespread in aging and evident from middle age [5,27]. An extensive literature review has shown distinct and reproducible patterns of atrophy including concentrations in the prefrontal and frontal cortex and medial temporal lobes, which have been associated with executive and memory declines also evident in healthy aging [7,8]. Cortical thinning appears to occur on an anterior–posterior axis, with the greatest rates of atrophy occurring in the frontal lobes [27]. This converges with some evidence from voxel-based morphometry [29], although volume and thickness measurements have produced contradictory results [28]. Lemaitre et al. (2012) systematically investigated regional and global patterns of brain morphometry. They aimed to clarify the different patterns of morphological changes that occur in aging by estimating cross-sectional changes in grey matter volume, cortical thickness and surface area in 216 healthy controls aged 18–87. All three measures showed age related reductions in the prefrontal cortex and this was accelerated compared to global levels of atrophy in cortical thickness and volume across the brain. There were more widespread changes in cortical thickness compared to volume with age, and surface area showed the least change with age. This suggests that surface area may not be a sensitive marker of morphological changes associated with brain aging, while cortical thickness might be the most sensitive [28].

Discrepancies between studies examining regional changes in brain structure in aging are likely due to the wide range of methods available to estimate brain morphometry, and the different statistical techniques used to model age effects [30]. Peelle et al. (2012) examined the impact of different image processing techniques and statistical models on estimates of age-related changes on 420 adults aged 18–77. Segmentation of the brain into its constituent tissues was improved by increasing the number of tissue classes and using a template more closely matched in age to the group studied [30]. The use of *total* grey matter as a covariate of no interest effected regional estimates of grey matter volume, and is an important consideration for the interpretation of group differences in estimates of brain morphometry [30]. Nevertheless, there is also a reassuring amount of consistency with regions of the frontal cortex, including the insulae, showing age related declines regardless of the image processing methods used or the statistical models applied [30].

It is now recognised that patterns of atrophy may reflect alterations in large scale network function. Regional atrophy patterns may be the result of degeneration in large-scale networks that also underlie the cognitive functions that decline with age [8,31]. As a result, a number of studies have used structural covariance methods to examine morphological changes across the lifespan within known functional or neurocognitive networks [31,32]. The morphology of primary sensory and motor networks seems to remain relatively preserved in aging compared to higher cognitive networks [31]. Higher cognitive networks including the default mode and executive control network seem to shift from more distributed to a more localised topology with aging [31]. Interestingly, there were notable reductions in connectivity with the posterior cingulate, a major hub of the default mode network (DMN) that is implicated in pathological aging.

Spreng et al. (2013) demonstrated declines in structural covariance in the default mode network in healthy aging that was more extensive in those who converted from mild cognitive impairment to Alzheimer’s disease (AD) and more still in the AD cohort. The findings support a network-based view of neurodegeneration in which the pattern of atrophy mirrors the intrinsic functional DMN [33]. Meunier et al. (2014) found that decreased grey matter integrity was associated with decreased regional connectivity of the language network but overall increased language network connectivity. This increased connectivity was thought to reflect reorganization to maintain performance, but was ultimately less efficient, by recruiting new regions to compensate for those within the network with reduced activity due to grey matter loss [34].

A number of studies using different methods of examining atrophy within networks have come to similar conclusions that distributed structural covariance networks shift to a more localised topology with age. In a study examining structural network integration based on cortical thickness in 102 young and 97 older adults, Chen et al. (2011) demonstrated a reduced modularity in the older compared to the younger group that they postulate reflects reduced functional integration in aging. Reduced modularity was seen in executive and default mode networks that likely underlie the cognitive deficits seen in aging [35]. Montembeault et al. (2012) partially replicated this finding using grey matter volume covariance. They demonstrated reduced structural covariance with aging in the executive and default mode networks, as well as a semantic network [8]. Finally, across 374 adults aged 64–68 and 428 adults aged 44–48, Zhu et al. (2012) showed lower global efficiency but higher local clustering in the older age group [36]. There appears to be some consensus then, across methods and measures of morphometry, for a loss of distributed structural covariance and modularity with aging and a shift to more localised covariance structures.

This raises the question as to what drives structural covariance network changes in healthy aging. Across the lifespan, there is evidence that networks show increasingly distributed structural covariance through development, with covariance becoming more localised in healthy aging [32]. Reductions in distributed structural covariance in aging may reflect reduced white matter connectivity between regions, and therefore reduced transmission of information, or co-activation of regions resulting in a divergence in the features of their morphometry over time [37].

## 4. Network-Based Neurodegeneration in Stroke

Stroke may initiate or aggravate neurodegeneration beyond that seen in healthy aging, and this neurodegeneration may be associated with cognitive impairment [38]. Cognitive impairment is common after ischaemic stroke, and dementia occurs in 15–30% of stroke patients within five years [39]. The effects of stroke are seen well beyond the site of damage [40], in seemingly healthy cortex, suggesting network wide effects of ischaemic stroke. There are focal grey matter volume changes that are associated with cognitive impairment after stroke [41]. Stebbins et al. (2008) compared grey matter volume between ischaemic stroke patients with no cognitive impairment and those with impairment in at least one domain. The thalamus seemed to be particularly vulnerable to degeneration after stroke, and its residual volume was associated with the presence of cognitive impairment in at least one cognitive domain. Other cortical regions also showed evidence of grey matter volume loss, although to a lesser extent [41]. Atrophy in the thalamus, irrespective of stroke location, and its association with multi-domain cognitive impairment, likely reflects the thalamus’s role as a multi-network hub [42]. Because of the vast interconnectedness of the thalamus, a stroke in any number of regions is likely to affect a network with thalamic involvement.

The concept of diaschisis, describing the remote effects of focal brain lesions, is over 125 years old [40]. There is a renewed interest in diaschisis in light of the discovery of the complex functional and structural network organisation of the brain. The terms connectional and connectomal diaschisis have been coined to describe the functional and structural changes that occur after damage within these complex brain networks [40]. Although the concept of diaschisis is most often applied to remote functional changes after stroke, it may also go some way to explaining remote changes in structure, including brain atrophy in regions distant from the stroke site. Regions remote from the lesion site may degenerate due to the loss of input from the lesioned site.

Brain atrophy is also evident in subcortical ischaemic vascular disease, with an increased white matter lesion burden associated with increased brain atrophy and cognitive decline [43]. The relationship between cognitive decline and white matter lesion load is mediated by brain volume loss, suggesting that atrophy plays a more direct role in cognitive decline than lesion burden [43,44]. However, the pathway from the increased burden of subcortical white matter lesions to brain volume loss is still unclear. Brain volume loss is often examined globally, not considering regional changes or white matter volume loss. Disruptions to white matter connectivity resulting from increasing white matter lesions may lead to denervation of cortical regions, which in turn leads to brain atrophy [43,45]. A similar pathway may also explain remote neurodegenerative effects of focal ischaemic stroke on cortical morphology.

A useful model for untangling the effects of lesion burden and brain atrophy is the study of cerebral autosomal dominant arteriopathy with subcortical infarcts and leukoencephalopathy (CADASIL). Patients with CADASIL can be prospectively studied with the expectation that new lesions are likely to occur as a result of their inherited small vessel disease. Patients are also younger than those with typical subcortical ischaemic vascular disease, allowing for more control over comorbidities. Duering et al. (2012) estimated cortical thickness in areas with white matter connections to incident subcortical infarcts in CADASIL patients. Cortical regions with a high probability of white matter connectivity to the incident infarct showed the greatest degree of focal cortical thinning [45]. This provides direct evidence of focal cortical thinning and remote secondary degeneration as a result of subcortical infarcts [45].

Studies of brain atrophy after stroke have so far been limited to regional volume changes and cross-sectional studies. Far fewer studies have examined the network level degenerative effects of stroke or the longitudinal effects, especially in comparison to the literature regarding ageing. Mirroring the default mode network degeneration shown in healthy aging, mild cognitive impairment (MCI) and Alzheimer’s disease (AD) [33], there is evidence of atrophy within the DMN in aging that is more extensive after ischaemic stroke [46]. Adapting the structural covariance method to examine correlations in the rate of atrophy across the brain (Figure 1b), Veldsman et al. (2017) demonstrated increased correlated atrophy after ischaemic stroke that suggests pathological network-wide degeneration similar to that seen in MCI and AD.

How does ischaemic stroke result in remote or network-wide neurodegenerative effects? Like the processes seen in healthy aging, disruption to white matter connectivity may result in a loss of cortical input that leads to the atrophy of previously connected areas. Alternatively, stroke may induce an ischaemic cascade that initiates or aggravates neurodegenerative processes that lead to brain atrophy. The pathway is far more complex in stroke than in healthy aging, because there is a background of vascular risk factors that likely led to stroke. This makes it difficult to separate the effects of an ischaemic stroke from accelerated atrophy occurring on the background of long-term vascular risk factors and mixed pathologies. Longitudinal studies are needed to identify changes in cortical morphology as a result of stroke, taking into account natural aging processes and the existing vascular burden.

## 5. Future Directions and Methodological Considerations 

If the brain is viewed as a complex network of functionally and structurally interconnected regions, then it is not surprising that changes associated with aging and disruption caused by stroke would be seen on a network-wide level and not within isolated regions. Brain atrophy is a macroscale indicator of neurodegeneration and is a strong predictor of cognitive impairment [43,47]. Neurodegenerative diseases target intrinsic functional networks, and atrophy mirrors healthy structural covariance networks, suggesting that meaningful patterns of atrophy underlie the variable cognitive phenotypes of dementia subtypes [4,12]. Examining covariation in morphological measures across the brain, and their disruption in disease, is becoming more popular in the aging literature, but it is still in its infancy in the stroke literature. Understanding the clinical significance, or determining the patterns of atrophy after stroke is complicated by the comorbid vascular burden and risk factors that predispose individuals to stroke in the first place. Nevertheless, there is an emerging picture of network-wide effects of focal ischaemic stroke that warrants further investigation, especially given the prevalence of cognitive impairment and dementia after stroke [38]. The data are relatively easy to acquire, requiring non-invasive structural scans which are often already obtained in scanning paradigms and stroke protocols. This makes the method appealing for clinical researchers and easily amenable to multi-centre research studies or for collection in open-data repositories.

Ongoing advances in imaging acquisition methods and analysis will allow for increasingly precise characterisation of morphological changes in the brain in aging and stroke. One of the most promising developments in the imaging of brain structure and brain atrophy is ultra-high field imaging. The relatively recent introduction of 7 tesla (T) scanners at research centres around the world provides better image contrast and higher spatial resolution. Many subcortical structures, such as the thalamic nuclei, basal ganglia and hippocampal substructures, can be more precisely delineated, and therefore atrophy can be more precisely quantified, than at 3T [48]. It is likely to be some time before 7T scanners become as widely available as 3T scanners, and there are still concerns regarding patient safety, contraindications and tolerance at the higher magnetic field strength of 7T [49]. This is particularly limiting for the elderly and for patient populations who are more likely to have had multiple surgeries, implants or other contraindications than for young, healthy volunteers. Nevertheless, the increased resolution will afford much better understanding of brain structure and its evolution over the course of the lifespan. Parallel acceleration sequences are increasing the resolution, signal-to-noise and contrast-to-noise ratios of structural scans at 3T [1], and are more easily acquired on patient populations. Multiple slices are acquired simultaneously, reducing the length of sequences, a major advantage for patients where scan time often has to be limited as their tolerance is often low.

Methods based on magnetic resonance imaging of the brain are subject to several sources of noise, including artefacts as the result of head motion. This is particularly problematic when comparing brain structure across groups in which group status may be correlated with the level of in-scan motion. For example, older participants may move more than younger participants, and patients may move even more still [1]. Visual inspection of structural images is an important quality control step, as large movement artefacts can often be seen clearly by the eye. MRI sequences have been developed to detect and correct for motion during the acquisition of the images [50,51]. This approach avoids having to discard scans post hoc due to motion artefacts.

As these methods are in their infancy, there is still significant work to be done in understanding their biological underpinnings. There is increasing evidence of their relationship to functional and structural networks [2,3]. For example, the same networks showing functional disruptions in prodromal Alzheimer’s disease show evidence of network-based atrophy [33]. However, it remains to be determined how these macroscale networks relate to histologically determined brain structure [21]. Nevertheless, structural covariance methods provide the potential to track the progression of atrophy, which can provide important prognostic information as well as quantitative evaluation of drug or rehabilitative interventions.
